# Diagnostic, Management, and Neonatal Outcomes of Colorectal Cancer during Pregnancy: Two Case Reports, Systematic Review of Literature and Metanalysis

**DOI:** 10.3390/diagnostics14050559

**Published:** 2024-03-06

**Authors:** Arianna Galante, Marco Cerbone, Francesco Mannavola, Marco Marinaccio, Luca Maria Schonauer, Miriam Dellino, Gianluca Raffaello Damiani, Vincenzo Pinto, Gennaro Cormio, Ettore Cicinelli, Antonella Vimercati

**Affiliations:** 1 Obstetrics and Gynaecology Unit, Department of Biomedical Sciences and Human Oncology, University of Bari “Aldo Moro”, 70124 Bari, Italy; ariannagalante94@gmail.com (A.G.);; 2 Division of Medical Oncology, Azienda Ospedaliera Universitaria Consorziale Policlinico di Bari, 70124 Bari, Italy; 3 Interdisciplinar Department of Medicine, University of Bari, 70124 Bari, Italy; 4 Gynecologic Oncology Unit, Istituto Tumori Bari Giovanni Paolo II IRCCS, 70124 Bari, Italy

**Keywords:** colorectal cancer, pregnancy, surgery, chemotherapy, systematic review

## Abstract

Objective: Colorectal cancer (CRC) during pregnancy is a rare occurrence, with a reported incidence of 0.8 cases per 100,000 pregnancies. Managing CRC during pregnancy poses substantial challenges for clinicians: the diagnosis is often complicated and delayed due to symptom overlap with pregnancy-related manifestations, and medical imaging is constrained by safety concerns for the foetus. Methods: This article presents two cases of advanced CRC diagnosed and managed during pregnancy. Additionally, we conducted a systematic review of the literature to assess diagnostic and prognostic factors involved in CRC in pregnant individuals. The systematic review, with pre-registration and approval through Prospero, involved an extensive search of medical databases (Pubmed, Web of Science, Scopus and Scholar) and statistical analysis using *t*-test for continuous variables and chi square for dichotomous variables. Results: A total of 1058 studies were identified. After applying exclusion criteria, sixty-six studies were included. Women whose initial symptoms were severe abdominal pain not responsive to common medical treatments and constipation (acute abdomen) had a mean gestational age at delivery lower than those who presented with paucisymptomatic onset. In our study groups, women who underwent chemotherapy during pregnancy had a higher mean gestational age at delivery and did not experience worse neonatal outcomes compared to those who did not undergo chemotherapy. Conclusions: CRC during pregnancy poses unique diagnostic and therapeutic challenges. Collaborative efforts among various medical disciplines are essential to manage CRC during pregnancy.

## 1. Introduction

Pregnancy-associated cancer (PAC) is an uncommon and challenging disease that raises crucial clinical, bioethical, and psychological issues, significantly impacting the health of pregnant women and the conceptus [1,2]. Due to bioethical concerns and the rarity of the disease, there is a lack of clinical trials and evidence-based guidelines. The epidemiology of PAC is challenging to study because nationwide registries often underestimate its incidence, databases are poorly comparable, and studies in the literature are heterogeneous because of different inclusion criteria and incoherent time intervals following delivery [3]. Approximately 1 in 2000 pregnancies are associated with a cancer diagnosis [4]. The most frequent cancers diagnosed during pregnancy are breast, melanoma, and cervical cancers, accounting for over 60% of all tumours diagnosed in pregnancy. Much rarer cancers in pregnancy include brain and nervous system tumours (9.3%), lymphomas (68%), thyroid cancer (3.8%), and colorectal cancer (CRC) (2.8%) [5]. Tumours of the gynaecological organs are rare [6]. Moreover, due to the absence of consensus and to the possible risks for the foetal health, treatment options for pregnant women with a diagnosis of cancer are limited: systemic treatment with chemotherapy should be avoided during the first trimester because it is associated with high risk of miscarriage and congenital malformations, but it can be administered during the second and third trimester. Surgery can be safely performed at any time during pregnancy. Radiotherapy should be postponed to the postpartum period regardless of the treated site due to the high risks for the foetus in terms of childhood cancer, foetal death, and mental retardation [7,8]. According to the Global Cancer Observatory-GLOBOCAN statistics, CRC is the second most common cancer in women, but it is a rare form of tumour during pregnancy [9]. In 2023, Sung et al. estimated 71,160 new cases of invasive CRC in American women, including 8990 diagnoses in women younger than 50 years [10]. Since 2010, the incidence of CRC has been increasing by approximately 3% per year in people under 50. We expect this number will continue to rise due to increasing cancer rates, delayed childbearing, and more sensitive screening. The new emerging factors, such as delayed childbearing and enhanced screening, contribute to the increased incidence in pregnant women [11]. Sporadic early onset CRC exhibits similar biological behaviour to later-onset disease. However, there are clinical distinctions [12]: early onset cancer is more likely to present with symptoms such as haematochezia (41%) and abdominal pain [13], with a predominance of left-sided tumours (73%) and advanced cases (27% with distant metastases). These characteristics were also observed in our patients [14]. Based on cancer registry data, survival rates are higher for early onset disease because younger patients are more likely to receive extensive treatments, including surgery and multi-agent adjuvant or neoadjuvant chemotherapies [15].

## 2. Case Reports

### 2.1. Case Report 1

**Figure 1:**
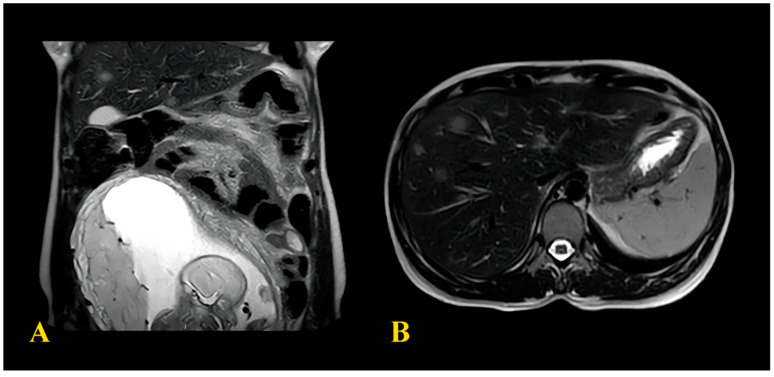
(**A**) MRI images showing the formation in amniotic sac; (**B**) CT image showing the hepatic metastases.

A 33-year-old patient with no significant medical history, prior surgical procedures, or family history of oncological disorders presented to the emergency room (ER) at Community Hospital of Barletta (Italy) due to severe abdominal pain unresponsive to common medications, constipation, and bloating (acute abdomen). The patient was 19 weeks pregnant with her second child; her first pregnancy had been uneventful. Obstetric assessment indicated no issues with the ongoing pregnancy. An abdominal Magnetic Resonance Imaging (MRI) revealed signs of intestinal obstruction suggestive of a sigmoid volvulus. She underwent urgent laparotomy with Hartmann sigmoid resection and colostomy the following day. Biopsy of the surgical specimen unveiled a sigmoid adenocarcinoma G2 causing strictures, ulceration, and full-thickness infiltration of the muscular layer with localised high-grade tumour and extension to the subserosal layer. No lymphatic, vascular, or perineural infiltration was detected. Surgical margins were tumour-free, as were the perivisceral lymph nodes. According to the histopathologic Tumour-Node Metastases staging system (pTNM) [14], the final stage was pT3bN0. Following surgery, a chest radiograph (RX) revealed no tumour-related findings, while an abdominal ultrasound identified a hepatic hyperechoic nodule (11 × 7 mm) in segments VII–VIII of uncertain nature. At 23 weeks of pregnancy, it was decided to transfer the patient to the Central Hospital “Policlinico di Bari” (Italy). Upon admission, her clinical condition was stable, with an Eastern Cooperative Oncology Group (ECOG) [16] performance status 1, with no abdominal pain, a functioning colostomy, normal sinus rhythm on electrocardiogram, and a foetus showing no distress. Blood tests indicated mild anaemia (haemoglobin 8.8 g/dL) but otherwise regular values for electrolytes, liver function, renal function, coagulation, platelets, and white blood cells. Tumour markers were assessed, showing an elevation of Alpha-fetoprotein (AFP) 109 ng/mL (normal values 0–7 ng/mL), while other tumour markers, including Carcinoembryonic antigen (CEA), Cancer Antigen 15.3 (CA15.3), Cancer Antigen 125 (CA125), and Cancer Antigen 19.9 (CA19.9), were within the normal range. During the hospitalisation, an abdominal MRI confirmed the presence of multiple hepatic nodules (diameter < 10 mm), suggestive of neoplastic lesions. The MRI, reported in (Figure 1), also showed a nodule on the parietal slope of the amniotic sac (20 mm) of unknown nature. This finding was not confirmed by ultrasound evaluation of the uterus.

Three hepatic biopsies were performed to ascertain the nature of these nodules, but the results were inconclusive. A multidisciplinary team comprising obstetricians, neonatologists, oncologists, and psychologists convened to discuss the case, deciding to initiate adjuvant chemotherapy during the pregnancy. Prior to discharge, the patient received a blood transfusion. Five cycles of folinic acid, fluorouracil, and oxaliplatin (FOLFOX) were administered biweekly (with a 25% dosage reduction as a precaution). Regular monitoring occurred every two weeks post-chemotherapy infusion. The foetus exhibited steady growth throughout treatment, with no signs of distress, and blood tests showed no haematological toxicity. The patient’s overall health remained favourable, and psychological support was provided. After the completion of chemotherapy, an abdomen ultrasound revealed reduced dimensions of the hepatic nodules. Subsequently, the multidisciplinary team scheduled an elective caesarean section (CS) after a three-week pause from the last cycle of chemotherapy at 37 weeks of pregnancy, with concomitant removal of the resectable hepatic nodules. The CS was successfully conducted at 37 weeks + 1 day, resulting in the birth of a 2940 g male infant. The newborn exhibited favourable conditions at birth, with arterial pH of 7.32, BE (B) of 0.8 mmol/L, BEecf of 1.7 mmol/L, and Apgar scores of 8 and 10. Routine assessments showed no distress. The histologic evaluation of hepatic nodules confirmed colic adenocarcinoma localisation. The histopathological analysis of the placenta was negative. After consulting with neonatologists regarding the benefits and risks of breastfeeding, the patient requested to inhibit lactation.

During hospitalisation, a contrast-enhanced chest-abdomen computer tomography (CT) demonstrated numerical and dimensional reduction of hepatic metastases. Given the positive response to chemotherapy, the multidisciplinary team decided to continue with FOLFOX (at full dosage), adding Bevacizumab, which was contraindicated in pregnancy. After six more cycles of chemotherapy, MRI and CT were performed, showing both numerical and dimensional regression of the hepatic nodules. In agreement with the general surgeons, it was decided to perform surgery to excise the liver metastasis and restore bowel continuity. The patient underwent a wedge resection involving segments VIII–V and IVb. After surgery, tumour marker values were within normal range. Given the favourable response to first-line chemotherapy, an additional six cycles of FOLFOX-BEVACIZUMAB were administered. One year post-diagnosis, the patient continues this therapeutic regimen, maintaining an optimal performance status (ECOG performance status 0). Additionally, her infant has reached one year of age, achieving normal developmental milestones.

### 2.2. Case Report 2

**Figure 2:**
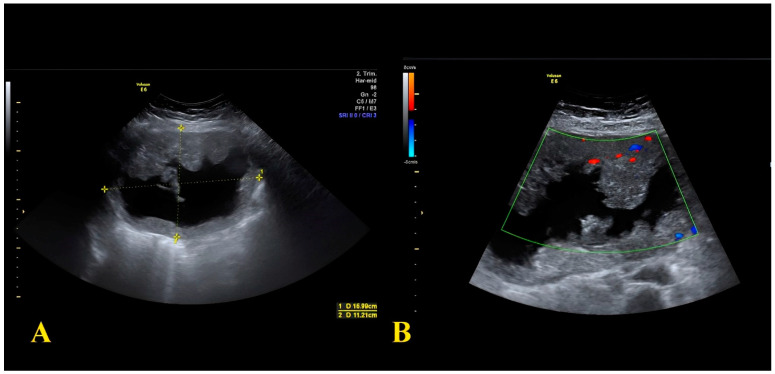
(**A**) Sonographic image showing the 16 × 11 cm adnexal cyst with a solid component; (**B**) Sonographic image showing intralesional vascularization.

A 43-year-old patient at her fourth pregnancy (preceded by three miscarriages), was admitted to the ER due to abdominal pain at 17 weeks of gestation. Her medical history included intestinal polyp removal via endoscopy and thyroid nodules with current euthyroidism. An abdominal ultrasound revealed a multilocular solid cyst in the right adnexa, exhibiting irregular internal margins and multiple septa and vegetations (Figure 2).

The cyst measured 15 cm in maximal diameter with a colour score of 3. Tumour markers assessment showed an elevation of AFP 100 ng/mL (normal range 0–7 ng/mL), CEA 14.6 ng/mL (normal range 0–5.2 ng/mL), and CA125 86.7 U/mL (normal range 0–35 U/mL), while CA19.9 and CA15.3 values were in the normal range. During hospitalisation, the patient underwent an MRI that indicated a sizable right adnexal lesion measuring 19 × 16 × 14 cm, characterised by protruding vegetations and a fluid component. There was not any free fluid in the abdomen nor significant lymphadenopathies. Pharmacological treatment of abdominal pain was unsuccessful; therefore, an urgent laparotomy was performed, during which the right adnexa was resected, alongside omental and peritoneal biopsies. Histological analysis identified an ovarian mucinous tumour with necrotic areas, while the fallopian tube remained tumour-free. Ki67 expression was 40%. Immunohistochemistry results indicated CK20 (+), PAX8 (−), CK7 (−), CDX2 (+), Estrogen Receptor (−), suggesting ovarian metastasis from gastrointestinal carcinoma. After the surgery, a colonoscopy highlighted a colon stenosis at the left flexure, which was biopsied.

Meanwhile, the pregnancy was monitored: foetal growth was regular, and there were no signs of foetal distress. The multidisciplinary team convened to formulate a management strategy: due to the advanced tumour stage and the poor performance status (ECOG 2), it was decided to wait until after the 28th week of gestation to perform a CS and a contextual intestinal resection. The CS was performed at 28 weeks of gestation, delivering a healthy newborn with favourable conditions at birth. Intraoperative inspection revealed multiple foci of cancer in the abdominal cavity, prompting a debulking surgery: left adnexa removal, excision of multiple parietal nodules, left hemicolectomy with colostomy placement, omentectomy, and cholecystectomy. Histopathological assessment unveiled a poorly differentiated adenocarcinoma from the large intestine, invading the serosal layer and perivisceral adipose tissue. Lymphatic, vascular, and perineural infiltration was detected. Twelve lymph nodes showed metastatic involvement. Metastasis were also detected in the omentum and peritoneum. The gallbladder was tumour-free. The final staging was, according to pTNM [17], pT4b, N2, M1b. Immunohistochemistry profiles indicated a mutated K-RAS, wild-type B-RAF and N-RAS, and no microsatellite instability. Following surgery, a contrast-enhanced whole-body CT revealed multiple hepatic metastases. The patient underwent adjuvant chemotherapy consisting of FOLFOX and Bevacizumab. After twelve cycles of chemotherapy, a CT indicated partial response, prompting a maintenance regimen involving 5-fluorouracil + Bevacizumab (five cycles). A subsequent positron emission tomography (PET) scan documented disease progression with multiple new metastases in the liver and lungs. Second-line therapy involving folinic acid, fluorouracil, irinotecan (FOLFIRI), and Aflibercept was recommended. Unfortunately, during this treatment course, the patient’s condition deteriorated, leading to her passing around two years after initial diagnosis.

## 3. Systematic Review and Metanalysis: Methods

This review was preregistered in the International Prospective Register of Systematic Reviews (PROSPERO) with protocol CRD42023440524 and is made in respect of the Preferred-Reporting-Items for Systematic Reviews and Meta-Analyses (PRISMA) guidelines [18].

### 3.1. Inclusion and Exclusion Criteria

Two different researchers scoped the existing literature, including studies that met the following inclusion criteria: (1) case reports; (2) written in the English language; (3) published in peer-reviewed journals; (4) with both an available abstract and full text; (5) involving women with a histologic diagnosis of colorectal cancer made during pregnancy; (6) involving women who underwent chemotherapy and/or surgery during pregnancy. We applied the following exclusion criteria: (1) scoping or systematic reviews; (2) literature not in the English language; (3) non-peer-reviewed papers; (4) papers with unavailable full text and abstract; (5) pregnant women with a histological diagnosis different from colorectal cancer; (6) women who were diagnosed or treated for colic cancer not during pregnancy; (7) papers without a histology diagnosis.

### 3.2. Search Strategy

We conducted searches in the Pubmed, Web of Science, Scopus, and Scholar databases, including a combination of the following terms with synonyms: “colon cancer”, “rectal cancer”, “colorectal cancer”, “pregnancy”, “management”, “surgery”, “maternal outcomes”, and “pediatric outcomes”. All searches were performed in English without a time limit. The initial search was completed in April 2023 and updated in June 2023.

### 3.3. Data Extraction

For each study, the two authors extracted the following data: authorship, year of publication, maternal and gestational age at the time of tumour diagnosis, presence of acute abdomen at the time of diagnosis, type of management (wait, upfront surgery, chemotherapy), type of delivery (caesarean, natural childbirth), neonatal outcomes including live birth, stillbirth, or miscarriage, neonatal major problems at the time of birth, and tumour staging based on the definitive histology. 

### 3.4. Quality of the Studies

**Figure 3:**
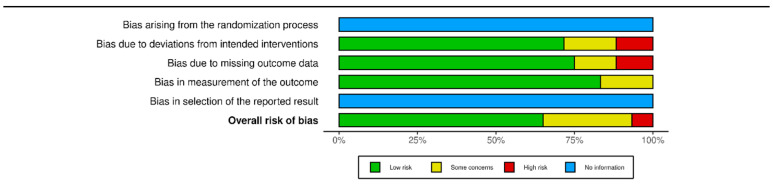
Summary plot of bias calculated with ROB2 tool via rob vis tool. Modified via [20].

The two authors independently evaluated the quality of the included studies using a modified ROB 2 scale [19], assessing five items and the overall risk of bias in the evaluations. The items evaluated were randomisation bias (not applicable), bias in deviation from intended intervention, bias due to missing outcome data, bias in measurement of outcome, and selection bias (not applicable). Each item was rated on a three-point scale: low, some concerns, high, or no information. The overall results are presented in (Figure 3).

## 4. Systematic Review and Metanalysis: Results

**Figure 4:**
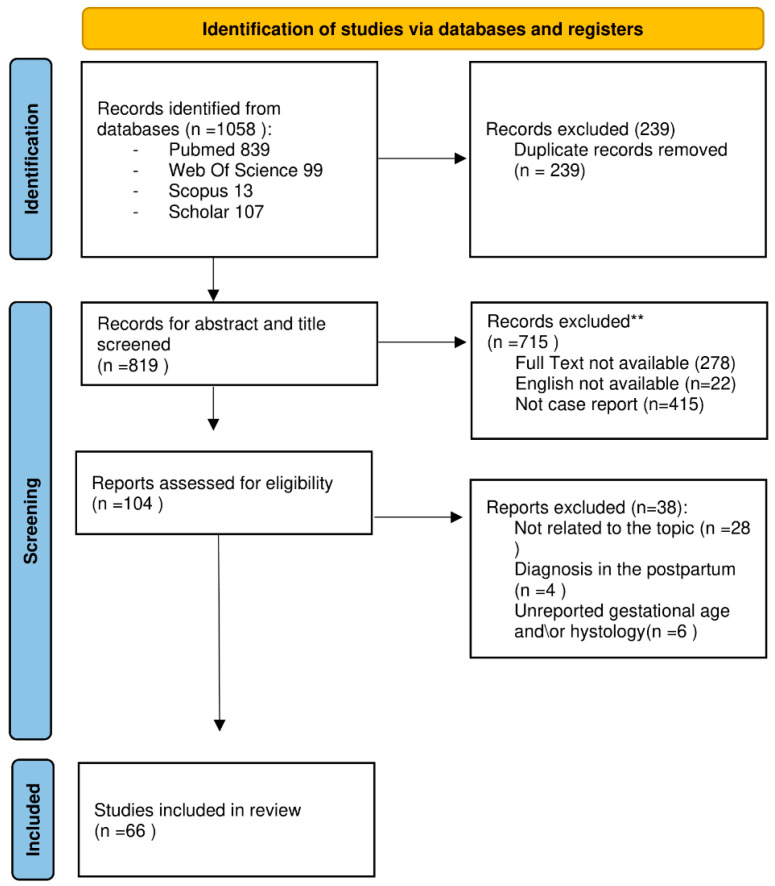
PRISMA flow diagram for new systematic review-FLOW CHART, modified from [21]. ** The authors manually excluded the records. No automation tools were used.

A total of 1058 studies were initially identified. Firstly, duplicates (*n*  =  239) were removed, resulting in 819 studies for abstract and title screening. Among these, 715 were excluded as they did not meet the inclusion criteria. Out of the remaining 104 studies eligible for full-text analysis, 28 were excluded because they were irrelevant to the topic, 6 due to unreported gestational age and histology, and 4 because the tumour diagnosis was made in the postpartum period. Consequently, 66 studies were ultimately included. See (Figure 4) for a summary of the selection process.

We enlisted 66 cases of colorectal cancer diagnosed during pregnancy. In (Table 1), we list the main characteristics of the study population.

### 4.1. Primary Outcomes

The mean age at diagnosis was 32.47 ± 4.17 years. The youngest girl diagnosed with PAC was 24 [30], while the oldest was 43 [27]. The mean gestational age at diagnosis was 25 weeks ± 8 weeks. The minimum gestational age was 9 weeks [26], and the maximum was 39 weeks [59]. The mean gestational age at birth was 32 weeks ± 5 weeks. The clinical presentation of patients was mostly non-specific. Acute abdomen at the time of diagnosis was observed in 53% of patients (*n* = 35). Upfront surgery was performed in 48.5% of patients (*n* = 32), while 39.9% (*n* = 27) underwent chemotherapy during pregnancy. The caesarean section was performed in 48.5% of patients (*n* = 32), while natural delivery occurred in 39.9% (*n* = 27). Data regarding the delivery modality were missing for 10.6% of patients (*n* = 7). The neonatal outcomes revealed live births in 75.8% of cases (*n* = 50), stillbirths in 7.6% of cases (*n* = 5), and miscarriages in 7.6% of cases (*n* = 5). In (Table 2), we list the main neonatal outcomes of the study population. The main histological finding was colic adenocarcinoma in 89.4% (*n* = 59) of cases. More rare histologic types included signet ring cell (*n* = 3) [37,50], undifferentiated (*n* = 3) [50,61], and neuroendocrine (*n* = 1) [50]. A total of 89.4% (*n* = 59) of the patients in the review were in an advanced stage (III/IV) at diagnosis. Among the patients who underwent upfront surgery, the laparotomic approach was the most commonly chosen method (96.8% of cases, *n* = 30), and 78% (*n* = 25) presented with an acute abdomen on the clinical onset. The delivery mode of women who underwent upfront surgery was caesarean section in 28.1% (*n* = 9) and vaginal delivery in 56.3% (*n* = 18). The delivery mode was not specified in 15.6% of patients (*n* = 5). There was no correlation found between mode of delivery and neonatal outcomes (*p* = 0.83). In (Table 3) we report a summary of the main findings of the review. 

### 4.2. Subgroup Analysis 1: Chemotherapy

In subgroup analysis 1, we differentiated two groups: women who underwent chemotherapy and women who did not. The first group had a mean gestational age at diagnosis of 22 ± 8 weeks, while the second group had a mean gestational age at diagnosis of 28 ± 6 weeks, with a statistically significant difference (*p* < 0.004, D Cohen 7.50, CI 95% 0.24–1.27). Regarding gestational age at delivery, women who underwent chemotherapy had a mean gestational age at delivery of 34 ± 4 weeks, while in the other group, it was 31 ± 5 weeks, but the difference was not statistically significant (*p* = 0.08). We calculated the differences in neonatal outcomes (live birth or stillborn/miscarriage) between the two groups and found no significant difference (*p* = 0.40). There was no significant difference in delivery modality between the two groups (*p* = 0.6).

### 4.3. Subgroup Analysis 2: Upfront Surgery

In subgroup analysis 2, we differentiated women who underwent upfront surgery from those who did not. The two sub-groups had no statistically significant difference in maternal age at diagnosis (*p* = 0.69) and gestational age at delivery (*p* = 0.43). However, there was a statistically significant difference in the median gestational age at diagnosis, 22 ± 8 weeks in women who underwent upfront surgery vs. 29 ± 7 weeks in women who did not (*p* < 0.001). We did not find a statistically significant difference between the two groups regarding neonatal outcomes (*p* = 0.29). We found a statistically significant difference (Chi-square 8.76, *p* < 0.003) between the two groups concerning the rate of caesarean sections: 66% (*n* = 18) of women who underwent upfront surgery had a spontaneous delivery. In contrast, only 28% (*n* = 9) of women who did not undergo upfront surgery had a spontaneous delivery.

### 4.4. Subgroup Analysis 3: Acute Abdomen at Diagnosis

In subgroup analysis 3, we differentiated women with acute abdomen at diagnosis vs. women with paucisymptomatic presentations. There was no statistically significant difference between the two groups’ gestational age and maternal age at diagnosis (*p* = 0.45, *p* = 0.25). However, there was a statistically significant difference between acute abdomen on onset and gestational age at birth (*p* = 0.04). Patients with acute abdomen on onset had deliveries at 31 weeks ± 6 weeks, while patients without acute abdomen on onset had a mean gestational age at delivery of 34 weeks ± 4 weeks. The difference between the means of the two groups was statistically significant (*p* < 0.04, Cohen d = 4.95, CI 95% 0.01–1.0). We calculated the differences in neonatal outcomes (live birth or stillborn/miscarriage) between the two groups and found a significant difference (Chi-square 3.85, *p* = 0.05). Among women with acute abdomen at diagnosis (*n* = 31), 25% (*n* = 8) had adverse neonatal outcomes. In contrast, among women with paucisymptomatic onset of symptoms (*n* = 29), only 6% (*n* = 2) had adverse neonatal outcomes. We did not find a statistically significant difference in the caesarean section rate between the two groups (*p* = 0.23).

### 4.5. Subgroup Analysis 4: Surgery versus Chemotherapy

In subgroup analysis 4, we differentiated patients who underwent surgery vs. patients who underwent chemotherapy. We found a significant difference in gestational age between the two groups at the time of the delivery (*p* < 0.03, CI 95% 0.4–8.7, Cohen d = 5.03). Patients who underwent surgery had a mean gestational age at the time of the delivery of 29 weeks ± 5 weeks, while patients who underwent chemotherapy had a mean gestational age at the time of delivery of 34 weeks ± 4 weeks. No other significant differences were found among the two groups of patients.

## 5. Discussion

### 5.1. Case Studies

Managing cancer during pregnancy is a complex task that requires a multidisciplinary approach involving oncologists, gynaecologists, paediatricians, and psychologists [70,71]. PAC diagnosis can be challenging for gynaecologists and primary care physicians and should adhere to current cancer guidelines applicable to general oncological patients [72,73]. Often, the typical signs and symptoms of cancer, such as changes in appetite, nausea, constipation, abdominal discomfort, pain, and fatigue, can be easily mistaken for pregnancy-related physiological changes [74]. This overlap in clinical presentation may contribute to delayed diagnosis and management of cancer during pregnancy [75,76]. In our cases, the patients were under 50 years old and had no family history of neoplastic diseases or significant medical/obstetrical personal history. Their pregnancies were physiological and under regular obstetrical check-ups and screenings. The cancer was diagnosed in both cases in the ER during the diagnostic work-up of acute abdominal pain. In case 1, the lesion causing acute pain was located in the bowel, and histologic findings indicated intestinal cancer with resection margins and perivisceral lymph nodes free of disease. Given the limited number of metastatic sites, the gestational age (25 weeks at the beginning of chemotherapy), and the favourable clinical conditions of the patient, chemotherapy with FOLFOX was initiated and continued at 75% dosage until she reached the term of the pregnancy. In case 2, the diagnosis was more challenging because the cyst removed during the urgent surgery was identified as mucinous cancer, with immunomorphological characteristics suggesting ovarian metastasis from gastrointestinal carcinoma. Subsequently, a colonoscopy revealed colon stenosis at the left flexure level, compatible with primary disease. The approach in case 2 differed significantly due to the earlier gestational age at the moment of the diagnosis (she was 17 weeks pregnant), the longer diagnostic process to find the primitive disease, and the more invasive disease. The multidisciplinary team decided to wait until the 28th week of pregnancy to deliver the foetus and initiate chemotherapy afterwards. The clinical approach was significantly different between the two cases, given that in case 1, the primary lesion was surgically removed in the initial surgery, and there were doubts about hepatic nodules, but the tumour was oligometastatic. In contrast, in case 2, only one metastasis was removed in the initial surgery, and imaging did not provide a clear picture of the actual extension of the disease. In case 1, chemotherapy was initiated during pregnancy, whereas in case 2, it was decided to wait until the 28th week to deliver the foetus and then re-evaluate the patient and start therapy. In both cases, we arranged caesarean sections simultaneous to the general neoplastic surgery. The newborns in both cases showed no clinical issues at birth and underwent regular neonatal follow-ups without any reported concerns.

### 5.2. Diagnostic Work-Up

The diagnostic work-up and staging of CRC during pregnancy is challenging; the importance of the information that invasive procedures, ionising radiation, and contrast agents can provide to the clinician should be carefully weighed against the risk for foetal health. Radiological procedures are categorised by the American College of Obstetricians and Gynaecologists (ACOG) into three main groups: very low dose (<0.1 mGy), low to moderate dose (<0.1–10 mGy), and higher dose (10.50 mGy) [75]. The maximum cumulative radiation is 100 mGys, defined as the threshold dose. While very low-dose procedures like chest radiography and mammography are considered safe, low-to-moderate dose procedures such as abdominal X-rays, chest CT, and lumbar spine radiography should be performed only when the benefits outweigh the risks to foetal health. In contrast, procedures associated with higher doses, including abdominal CT, pelvic CT, and whole-body PET/CT, should generally be avoided during pregnancy [77]. Contrast agents are generally discouraged during pregnancy due to the potential placental passage. However, the ACOG guidelines permit contrast use in specific situations where the diagnostic performance of imaging is expected to improve maternal-foetal outcomes. Among contrast agents, gadolinium appears safer, whereas iodinated contrasts are generally contraindicated due to a lack of studies in humans [78]. To mitigate the risks, ultrasonography and magnetic resonance imaging (MRI) techniques are preferred in pregnant women to avoid the exposition to ionising radiation. Serum tumour markers like CA 125, CA 15.3, AFP, and SCC are generally not significant during pregnancy, but markers like AMH, CEA, CA 19-9, and HE4 usually do not exhibit increased levels during pregnancy, making them potentially applicable [79]. In conclusion, chest X-rays with abdominal shielding and MRI imaging without contrast can be safely employed for staging oncologic patients during pregnancy. Serum tumour markers may be helpful in selected cases. The emerging popularity of cell-free DNA screening increases cancer diagnosis in asymptomatic pregnant women. Amant et al. reported three cases of cancer (ovarian cancer and two forms of lymphoma) among a cohort of 4000 pregnant asymptomatic women who underwent non-invasive prenatal testing (NIPT) [80]. These findings question the utility of NIPT tests like a cancer soft marker, but further studies are needed to assess the clinical reliability and diagnostic and prognostic value of this new approach [81].

### 5.3. Treatment Options: Upfront Surgery

Oncologic treatment during pregnancy may include surgery, especially after the first trimester, systemic treatment with medications compatible with pregnancy, and, in selected cases, radiotherapy. Radical surgery is one of the most effective strategies to improve cancer outcomes. In a 6018 cohort of second- and third-trimester pregnant women who underwent laparoscopic and laparotomic abdominal surgery, statistically significant (*p* < 0.001) benefits were observed in the laparoscopic group in terms of operative time, need for blood transfusion, and hospital stays compared to the laparotomy group [82]. Laparoscopy also results in fewer adverse foetal and maternal complications compared to laparotomy [83]. However, managing PAC can pose challenges, including an increased rate of severe maternal morbidity associated with cancer, often necessitating transfusions, hysterectomy, and ventilation complications [84,85].

### 5.4. Treatment Options: Chemotherapy

The placenta functions as a biological interface between the mother and the foetus, providing nutrients and oxygen and acting as an immunologic barrier [86,87]. Under physiological conditions, maternal and foetal blood are functionally connected but separated by the placental barrier, which regulates the passage of xenobiotics, including antineoplastic agents [88,89]. The transfer of anticancer agents across the placental barrier is influenced by their properties such as molecular weight, ionisation, binding to plasma proteins, and hydro-lipophilicity. Highly lipophilic molecules with a molecular weight of less than 500 Daltons, without ionisation, and bound to plasma proteins are likely to cross the placental barrier through passive diffusion, a process that does not require energy and is directly dependent on drug concentration in the maternal circulation [90,91]. Larger-molecular-weight hydrophilic molecules may be actively transported across the placental barrier by ATP-powered transporters against the concentration gradient, such as multidrug resistance protein, glycoprotein-p, and breast cancer resistance protein [92]. Antineoplastic toxicity depends mainly on the timing of exposure. Within the first ten days after conception, there is an “all-or-nothing” phenomenon, where the embryo’s survival depends on the extent of cell destruction [93]. The administration of antineoplastic drugs is generally contraindicated from 10 days after conception and until 14 weeks of pregnancy due to the high risk of major malformations (heart, neural tube, upper and lower limbs, eyes, palate, and ears) that may occur during the organogenesis [94]. According to the third consensus on gynaecological cancers, chemotherapy is contraindicated during the first trimester to avoid damage during organogenesis. Dosing of chemotherapeutic drugs during pregnancy should consider molecular weight, without significant differences in dosing compared to non-pregnant patients [6]. Chemotherapy is generally not recommended beyond 35 weeks of pregnancy. It is essential to allow a three-week interval between the last cycle of chemotherapy and delivery to allow maternal and foetal bone marrow to recover. The standard chemotherapy for advanced colorectal carcinoma is based on FOLFOX or FOLFIRI regimens (5-FU, leucovorin, and oxaliplatin or irinotecan) [95]. There are no specific guidelines for the management of colorectal cancer during pregnancy, but studies consider the FOLFOX and FOLFIRI regimens feasible during the second and third trimesters [96]. The human safety of 5-FU has been evaluated retrospectively through a literature review of case reports, case series, and surveys [97]. Exposure during pregnancy was associated with a 1% overall rate of major malformations when chemotherapy was administered during the second or third trimester, which was not significantly different from the rate in the general population. Platinum-based therapies, such as oxaliplatin, have also been associated with relatively safe foetal outcomes when administered in the second and third trimesters, with agents such as cisplatin and carboplatin demonstrating rates of major foetal malformation of 1% and 0%, respectively [97]. Oxaliplatin has been associated with an increased incidence of the foetal condition of small for gestational age [98]. Among four patients registered in the International Network on Cancer, Infertility, and Pregnancy database who received combination FOLFOX treatment in the second or third trimesters, all of them delivered healthy infants [99]. Two reports of irinotecan administered during the second trimester did not note any complications or malformations. Indeed, 5-FU is considered to not result in significant long-term disabilities, apart from the tendency for newborns to be smaller than those who are not exposed, and oxaliplatin and irinotecan have also been reported to be safe [46].

## 6. Conclusions

We presented these cases and the accompanying review to shed light on the complexities of managing PAC. We want to underscore the importance of early cancer diagnosis and individualised treatment plans: the collaboration of multidisciplinary healthcare specialists should be encouraged to optimise clinical outcomes for both pregnant women and foetuses.

The successful management of case 1, including upfront surgery and adjuvant chemotherapy during pregnancy, made it possible to reach the pregnancy term (37 weeks) and deliver a healthy newborn. This case demonstrates the effectiveness of a coordinated and well-executed healthcare strategy. Case 2 highlights that despite the multidisciplinary efforts, in some cases of advanced PAC, the aggressiveness of the disease and poor performance status of the patient can contraindicate invasive management of the disease. This case emphasises the importance of early detection and underscores the need for ongoing research and advancements in maternal-foetal medicine and oncology to improve outcomes in such complex cases.

Our systematic review provides clinically significant insights into the characteristics and management approaches for pregnant women diagnosed with colorectal cancer. We demonstrate the variability in presentation, treatment modalities, and outcomes. Gestational age at diagnosis, mode of delivery, and acute symptoms appear to influence maternal and neonatal outcomes. Our statistical analysis revealed the importance of early diagnosis of PAC: the cases where the diagnosis was delayed often led to upfront surgery due to acute abdomen, resulting in poor maternal and neonatal outcomes with higher caesarean rates and earlier gestational age at the delivery. In contrast, we found that women with an early diagnosis were more likely to undergo chemotherapy during pregnancy, resulting in better control of the disease and later gestational age at delivery. Despite the uncertainties about the effects of chemotherapy on the foetus, we did not find different neonatal outcomes between those exposed to chemotherapy during pregnancy and those not. Chemotherapy during pregnancy is a feasible and safe treatment for PAC, allowing the clinician to treat the maternal disease and concomitantly delay foetal delivery to a safer gestational age.

In the author’s current knowledge, this is one of the studies on PAC with the most significant sample size and longest time extension; our statistical analysis highlighted some topics of emerging clinical importance in managing pregnant women diagnosed with CRC. The study’s main limitations are the high heterogeneity of included articles, the low/moderate overall risk of bias, mainly because of unreported data, and the absence of long-term outcomes for both the patients and the newborns. Because of ethical concerns and the rarity of PAC, there is a lack of prospective or randomised studies on CRC during pregnancy; this causes a lack of evidence-based guidelines for managing the disease. Medical knowledge and multidisciplinary care advancements continue to offer new options to improve outcomes in these rare and intricate situations. Further efforts in oncological gynaecology are needed to refine our understanding and improve care for pregnant individuals facing this challenging disease.

## Figures and Tables

**Table 1:** Main characteristics of the population in the study.

Author	Year	Maternal Age at Diagnosis	Hystotype	Tumour Stage	Chemotherapy
Santana et al. [22]	2022	32	Colic adk	III	Not performed
Tarik et al. [23]	2022	41	Colic adk	IV	Adjuvant
Yang et al. [24]	2021	36	Undifferentiated	IV	Adjuvant
Maqueira et al. [25]	2020	30	Colic adk	IV	Not performed
Alkhamis et al. [26]	2020	30	Colic adk (focal)	0	Not performed
Frydenberg et al. [27]	2020	43	Colic adk	IV	NeoAdjuvant
Petruzzelli et al. [28]	2020	38	Colic adk	III	Adjuvant
Sravantthi et al. [29]	2020	31	Colic adk	III	Neo + Adjuvant
Ochoa et al. [30]	2020	24	Signet ring	IV	Not performed
Lee et al. [31]	2019	32	Colic adk	IV	Not performed
Lee et al. [31]	2019	25	Colic adk	IV	NeoAdjuvant
Lee et al. [31]	2019	34	Colic adk	IV	NeoAdjuvant
Munteanu et al. [32]	2019	36	Colic adk	IV	Not performed
Žegarac et al. [33]	2019	33	Colic adk	II	Not performed
Zegarac et al. [33]	2019	33	Colic adk	III	Not performed
Muntenau et al. [32]	2019	36	Colic adk	III	Adjuvant
Murphy et al. [34]	2018	36	Colic adk	IV	Not performed
Xu et al. [35]	2018	31	Colic adk	II	Not performed
Xu et al. [35]	2018	31	Colic adk	IV	Adjuvant
Murphy et al. [34]	2018	36	Colic adk	IV	Adjuvant
Makhijani et al. [36]	2017	29	Colic adk	III	Adjuvant
Wenfeng Ye et al. [37]	2017	29	Signet ring	IV	Not performed
Jones et al. [38]	2017	33	Colic adk	III	Not performed
Jones et al. [38]	2017	33	Colic adk	III	Adjuvant
Makhjani et al. [36]	2017	29	Colic adk	IV	Neo + Adjuvant
Gabriel et al. [39]	2016	31	Colic adk	IV	Not performed
Ossendorp et al. [40]	2016	34	Colic adk	IV	Not performed
Ossendrorp et al. [40]	2016	34	Colic adk	IV	Adjuvant
Makoshi et al. [41]	2015	33	Colic adk	IV	Adjuvant
Chouaib et al. [42]	2015	32	Neuroendocrine		Not performed
P Kocián et al. [43]	2015	37	Colic adk	0	Not performed
Makoshi et al. [41]	2015	33	Colic adk	III	Neo + Adjuvant
Dogan et al. [44]	2013	38	Colic adk	IV	NeoAdjuvant
HO et al. [45]	2012	29	Colic adk	III	Not performed
HO et al. [45]	2012	29	Colic adk	III	Adjuvant
Jeppesen et al. [46]	2011	26	Colic adk	IV	Adjuvant
Jeppesen et al. [46]	2011	26	Colic adk	III	Neo + Adjuvant
Gensheimer et al. [47]	2009	25	Colic adk	IV	Adjuvant
Kanate et al. [48]	2009	40	Colic adk	IV	NeoAdjuvant
Taylor et al. [49]	2009	34	Colic adk	IV	Adjuvant
Taylor et al. [49]	2009	34	Colic adk	III	Adjuvant
Duffy et al. [50]	2008	33	Signet ring	IV	Not performed
Duffy et al. [50]	2008	33	Mixed	IV	Adjuvant
Mechery et al. [51]	2007	34	Colic adk	IV	Not performed
Lolis et al. [52]	2007	29	Colic adk	III	Not performed
Lolis et al. [52]	2007	29	Colic adk	III	Adjuvant
Chêne et al. [53]	2006	26	Colic adk	III	Not performed
Penney et al. [54]	2006	34	Colic adk	IV	Not performed
Harma et al. [55]	2005	30	Colic adk	IV	Not performed
Minter et al. [56]	2005	28	Colic adk	II	Not performed
Papathanasiou et al. [57]	2004	37	Colic adk	IV	Not performed
S Kömürcü et al. [58]	2001	33	Colic adk	IV	Not performed
Kitoh et al. [59]	1998	34	Colic adk	IV	Not performed
Kitoh et al. [59]	1998	35	Colic adk	IV	Not performed
Rojansky et al. [60]	1997	39	Colic adk	III	Not performed
Vitoratos et al. [61]	1994	32	Undifferentiated	IV	Not performed
Heres et al. [62]	1993	38	Colic adk	IV	Not performed
Yoshinobu et al. [63]	1993	27	Colic adk	III	Not performed
Heise et al. [64]	1992	29	Colic adk	III	Adjuvant
Gonsoulin et al. [65]	1990	33	Colic adk	IV	Not performed
R Jaffe et al. [66]	1989	42	Colic adk		Not performed
Tsukamoto et al. [67]	1986	29	Colic adk	IV	Not performed
Nesbitt et al. [68]	1985	35	Colic adk	III	Not performed
Nesbitt et al. [68]	1985	31	Colic adk	III	Not performed
Nesbitt et al. [68]	1985	28	Colic adk	IV	Not performed
Hill et al. [69]	1984	29	Colic adk	IV	Not performed

Legenda: Adj: adjuvant chemotherapy, Neo: neoadjuvant chemotherapy, Adk: adenocarcinoma.

**Table 2:** Main data on neonatal outcome of the population in the study.

Author	Year	Gestational Age at Diagnosis (Weeks)	Mode of Birth	Gestational Age at Birth	Neonatal Outcomes
Santana et al. [22]	2022	28	CS	32	Liveborn
Tarik et al. [23]	2022	24	CS	28	
Yang et al. [24]	2021	39	CS	39	Liveborn
Maqueira et al. [25]	2020	35	VB	35	Liveborn
Alkhamis et al. [26]	2020	9	VB	40	Liveborn
Frydenberg et al. [27]	2020	23	CS	34	Liveborn
Petruzzelli et al. [28]	2020	26	VB	37	Liveborn
Sravantthi et al. [29]	2020	21			
Ochoa et al. [30]	2020	22	VB	25	Liveborn
Lee et al. [31]	2019	37	VB	37	Liveborn
Lee et al. [31]	2019	19	VB	33	Stillbirth
Lee et al. [31]	2019	11	VB	36	Liveborn
Munteanu et al. [32]	2019	33	CS	33	Liveborn
Žegarac et al. [33]	2019	22	VB	26	Liveborn
Zegarac et al. [33]	2019	22			
Muntenau et al. [32]	2019	33	CS	33	Liveborn
Murphy et al. [34]	2018	32	CS	35	Liveborn
Xu et al. [35]	2018	33	CS	33	Liveborn
Xu et al. [35]	2018	33	CS	33	Stillbirth
Murphy et al. [34]	2018	33	CS	35	Stillbirth
Makhijani et al. [36]	2017	17			
Wenfeng Ye et al. [37]	2017	27	CS	28	Liveborn
Jones et al. [38]	2017	29	VB	29	Liveborn
Jones et al. [38]	2017	29	VB	23	Abortion
Makhjani et al. [36]	2017	17			
Gabriel et al. [39]	2016	30	CS	35	Liveborn
Ossendorp et al. [40]	2016	32	CS	33	Liveborn
Ossendrorp et al. [40]	2016	32	CS	33	Liveborn
Makoshi et al. [41]	2015	11	VB	38	Liveborn
Chouaib et al. [42]	2015	32	VB		Liveborn
P Kocián et al. [43]	2015	27	CS	28	Liveborn
Makoshi et al. [41]	2015	11	VB	38	Liveborn
Dogan et al. [44]	2013	19	CS	36	Liveborn
HO et al. [45]	2012	21	VB	37	Liveborn
HO et al. [45]	2012	21	VB	37	Liveborn
Jeppesen et al. [46]	2011	10	CS	33	Liveborn
Jeppesen et al. [46]	2011	11	CS	23	Abortion
Gensheimer et al. [47]	2009	12	VB	33	Liveborn
Kanate et al. [48]	2009	23	CS	31	Liveborn
Taylor et al. [49]	2009	15	VB	37	Liveborn
Taylor et al. [49]	2009	15	VB	38	Liveborn
Duffy et al. [50]	2008	30	CS	33	Liveborn
Duffy et al. [50]	2008	30	CS	33	Liveborn
Mechery et al. [51]	2007	38	VB	38	Liveborn
Lolis et al. [52]	2007	27	CS	33	Liveborn
Lolis et al. [52]	2007		CS	33	Liveborn
Chêne et al. [53]	2006	22	CS	34	Liveborn
Penney et al. [54]	2006	19			
Harma et al. [55]	2005	37	VB	37	Liveborn
Minter et al. [56]	2005	16	VB	16	Abortion
Papathanasiou et al. [57]	2004	34	CS	34	Liveborn
S Kömürcü et al. [58]	2001	30	CS	34	Liveborn
Kitoh et al. [59]	1998	39	CS	39	Liveborn
Kitoh et al. [59]	1998	31	CS	35	Liveborn
Rojansky et al. [60]	1997	34	VB	34	Liveborn
Vitoratos et al. [61]	1994	26	VB	26	Stillbirth
Heres et al. [62]	1993	27	CS	32	Liveborn
Yoshinobu et al. [63]	1993	19	VB		Liveborn
Heise et al. [64]	1992	35	CS		Liveborn
Gonsoulin et al. [65]	1990	25	CS	25	Stillbirth
R Jaffe et al. [66]	1989	30	VB	30	Liveborn
Tsukamoto et al. [67]	1986	21		21	Abortion
Nesbitt et al. [68]	1985	22		22	Abortion
Nesbitt et al. [68]	1985	25	CS	32	Liveborn
Nesbitt et al. [68]	1985	29	VB	29	Liveborn
Hill et al. [69]	1984	26	VB	26	Liveborn

Legenda: CS: caesarean section; VB: vaginal birth.

**Table 3:** Summary of main findings of the systematic review.

**Maternal Data**		
Mean age at diagnosis (years)	32.47 ± 4.17	
Mean gestational age at diagnosis (weeks)	25 ± 8	
Mean gestational age at birth (weeks)	32 ± 5	
**Type of Surgery**	%	*n*
Laparotomy	47.0	31
Laparoscopy	0	0
Endoscopic treatment	1.5	1
Not performed	39.4	26
Missing data	12.1	8
**Chemotherapy during Pregnancy**	%	*n*
Total	39.9	27
Adjuvant	27.3	18
Neoadjuvant	7.6	5
Neoadjuvant + Adjuvant	6.1	4
Not performed	59.1	39
**Histology**	%	*n*
Colic adenocarcinoma	89.4	59
Signet ring cell	4.5	3
Undifferentiated	4.5	3
Neuroendocrine	1.5	1
**Tumour Stage**	%	*n*
I	3.0	2
II	4.5	3
III	31.8	21
IV	57.6	38
missing	3.0	2
**Mode of Delivery**	%	*n*
Caesarean section	48.5	32
Natural delivery	39.9	27
Missing data	10.6	7
**Neonatal Outcomes**	%	*n*
Live births	75.8	50
Stillbirths	7.6	5
Miscarriages	7.6	5
Missing data	9.0	6

## Data Availability

All data are reported in the text.
